# The Inhibitory Activity of Anthraquinones against Pathogenic Protozoa, Bacteria, and Fungi and the Relationship to Structure

**DOI:** 10.3390/molecules25133101

**Published:** 2020-07-07

**Authors:** Mendel Friedman, Alexander Xu, Rani Lee, Daniel N. Nguyen, Tina A. Phan, Sabrina M. Hamada, Rima Panchel, Christina C. Tam, Jong H. Kim, Luisa W. Cheng, Kirkwood M. Land

**Affiliations:** 1 Healthy Processed Foods Research Unit, Agricultural Research Service, United States Department of Agriculture, Albany, CA 94710, USA; 2 Department of Biological Sciences, University of the Pacific, Stockton, CA 95211, USA; a_xu2@u.pacific.edu (A.X.); r_lee50@u.pacific.edu (R.L.); d_nguyen62@u.pacific.edu (D.N.N.); t_phan18@u.pacific.edu (T.A.P.); s_hamada@u.pacific.edu (S.M.H.); r_panchel1@u.pacific.edu (R.P.); kland@pacific.edu (K.M.L.); 3 Foodborne Toxins Detection and Prevention Research Unit, Agricultural Research Service, United States Department of Agriculture, Albany, CA 94710, USA; christina.tam@usda.gov (C.C.T.); jongheon.kim@usda.gov (J.H.K.); luisa.cheng@usda.gov (L.W.C.)

**Keywords:** *Trichomonas vaginalis*, *Tritrichomonas foetus*, cell assays, trichomoniasis, trichomonosis, anthraquinones, structure–activity relationships, inactivation, mechanisms, health benefits, research needs

## Abstract

Plant-derived anthraquinones were evaluated in cell assays for their inhibitory activities against the parasitic protozoa *Trichomonas vaginalis* human strain G3 that causes the sexually transmitted disease trichomoniasis in women, *Tritrichomonas foetus* bovine strain D1 that causes sexually transmitted diseases in farm animals (bulls, cows, and pigs), *Tritrichomonas foetus*-like strain C1 that causes diarrhea in domestic animals (cats and dogs), and bacteria and fungi. The anthraquinones assessed for their inhibitory activity were anthraquinone, aloe-emodin (1,8-dihydroxy-3-hydroxymethylanthraquinone), anthrarufin (1,5-dihydroxyanthraquinone), chrysazin (1,8-dihydroxyanthraquinone), emodin (1,3,8-trihydroxy-6-methylanthraquinone), purpurin (1,2,4-trihydroxyanthraquinone), and rhein (1,8-dihydroxy-3-carboxyanthraquinone). Their activities were determined in terms of IC_50_ values, defined as the concentration that inhibits 50% of the cells under the test conditions and calculated from linear dose response plots for the parasitic protozoa, and zone of inhibition for bacteria and fungi, respectively. The results show that the different substituents on the anthraquinone ring seem to influence the relative potency. Analysis of the structure–activity relationships in protozoa indicates that the aloe-emodin and chrysazin with the highest biological activities merit further study for their potential to help treat the diseases in women and domestic and farm animals. Emodin also exhibited antifungal activity against *Candida albicans*. The suggested mechanism of action and the additional reported beneficial biological properties of anthraquinones suggest that they have the potential to ameliorate a broad spectrum of human diseases.

## 1. Introduction

**Figure 1:**
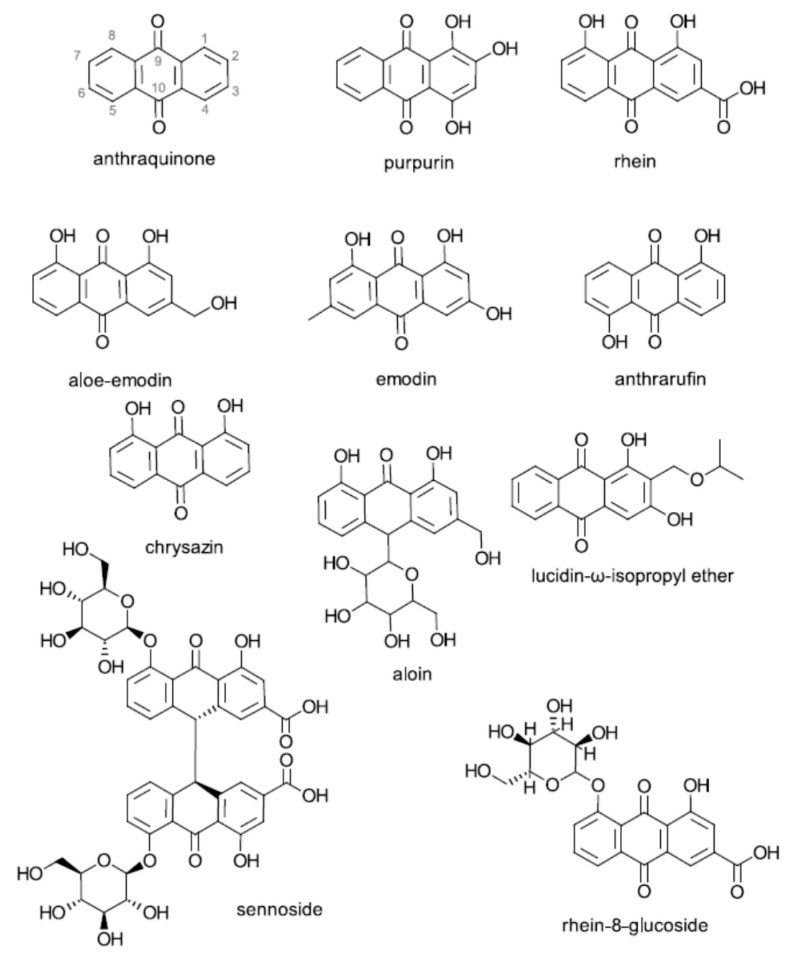
Chemical structures of anthraquinone and derivatives.

A number of plant species (including *Rheum palmatum* L., *Cassia obtusifolia* L., and multiple *Rubia* species) are reported to biosynthesize anthraquinone derivatives [1,2,3]. The biosynthesis of anthraquinones occurs via the polyketide and shikimic acid pathways [4]. Anthraquinones are aromatic compounds with a 9,10-dioxoanthracene core substituted in the two benzene rings with phenolic OH and aliphatic groups that have been reported to have beneficial biological properties. Figure 1 shows the structures of the seven compounds evaluated against trichomonads in the present study: anthraquinone; anthrarufin (1,5-dihydroxy-9,10-anthraquinone); chrysazin (1,8-dihydroxy-9,10-anthraquinone); purpurin (1,2,4-trihydroxy-9-10-anthraquinone); emodin (1,3,8-trihydroxy-6-methyl-9,10-anthraquinone); aloe-emodin (1,8-dihydroxy-3-hydroxymethyl-9,10-anthraquinone), and rhein from the edible rhubarb plant. The structure of a new anthraquinone (lucidin-isopropyl ether) that has strong anti-*Trichomonas vaginalis* activity, recently isolated from the roots of *Morinda panamensis* Seem., is also shown in Figure 1 [5]. Anthraquinones occur naturally in the plant as glycosides, as illustrated in Figure 1 by the structures of sennoside and rhein-8-glucoside. It seems, however, that aglycones produced on removal of the carbohydrate side chains are used in many reported studies, including this one [6].

Interest in anthraquinones arises from the fact that they are reported to have multiple potential health benefits in cells and in vivo, including antibiotic [1,4,7,8], anticancer [2,9,10,11], antidiabetic [4], antifungal [12], anti-inflammatory [13], anti-obesity [14], antioxidative [13,15], antiprotozoal [16,17,18], antiviral [19], cardioprotective [20], hepatoprotective [21], and neuroprotective [22] properties. Emodin and aloe-emodin are already widely used as stimulant laxatives sold in pharmacies to treat constipation [6,23,24].

*Trichomonas vaginalis* is the most common non-viral sexually transmitted human venereal disease that is most often detected in women and that does not appear to decrease with age, reaching a maximum rate in 48- to 51-year old women [25]. *Tritrichomonas foetus* protozoa strains cause the related sexually transmitted disease bovine trichomoniasis in cattle and pigs [26,27], and intestinal diarrhea in domestic cats and dogs [28,29,30]. The most cost-effective method to control the disease is often culling the infected animals. In domesticated cats, the disease is transmitted by the oral–fecal route where it infects the gastrointestinal tract, causing persistent diarrhea. Because drugs used to treat these diseases, such as metronidazole, show reduced potency because of resistance acquired by the pathogens [31], there is a need to develop new therapies [32].

We previously reported on the anti-oxidative and anti-inflammatory activities of four anthraquinones in chemical and cell assays [13] and on the anti-obesity properties of purpurin in mice on a high-fat diet [14]. In several publications related to the present study, we reported that the tomato glycoalkaloid tomatine [33], theaflavin-rich black tea extracts [34], potato-peel-containing glycoalkaloids and phenolic compounds [35] inhibited the growth of *T. vaginalis* human strain G3, *T. foetus* bovine strain D1, and *T. foetus* feline strain C1. We also reported that the potato peels and glycoalkaloids exhibited strong anti-obesity properties in mice on a high-fat diet [36].

In view of the bioactivities of anthraquinone compounds in cell assays and in vivo, and the anti-parasitic trichomonad properties of structurally different plant compounds, selected anthraquinones are investigated here for their ability to inhibit the growth of the three pathogenic trichomonad strains. This study aims, therefore, to use cell assays to compare the structure–activity relationships of the above-mentioned seven plant-derived anthraquinones against three parasitic protozoa strains that cause sexually transmitted diseases in humans, farm and domestic animals as well as some bacterial and fungal pathogens. The results suggest that the number, nature, and location of substituents on the anthraquinone moiety strongly influence their inhibitory potencies against the pathogenic protozoa and fungi.

## 2. Results and Discussion

The IC_50_ values (concentration that inhibits 50% of three pathogenic protozoa under the test conditions) were calculated from non-linear dose–response plots using the GraphPad software (San Diego, CA, USA) and as described previously [34].

The data in Table 1 show that, for *T. vaginalis* G3 (human), when comparing the lowest IC_50_ value (highest activity) for aloe emodin (0.6109 µM) to the highest IC_50_ value (lowest activity) for purpurin (101.6 µM), aloe emodin has 166.2 times greater inhibitory activity than purpurin. Similar calculations of the ratios of the other compounds (compared to purpurin) using IC_50_ values shown in Table 1 indicate that rhein, chrysazin, and anthraquinone are 20.2, 11.4, and 9.8, respectively. The IC_50_ values for anthrarufin and emodin were not determined. The data show that for the human trichomonad, aloe-emodin exhibited the highest activity among the five evaluated compounds followed by chrysazin.

For the *T. foetus* (feline) strain, Table 1 shows that, in terms of IC_50_ value, chrysazin is 137.8 times more active than purpurin. The corresponding ratios (enhanced activities compared to purpurin) for aloe-emodin, rhein, anthrarufin, and anthraquinone are 94.4, 33.8, 16.4, and 5.2, respectively. The IC_50_ value for emodin was not determined. These data show that for the feline trichomonad strain, chrysazin shows the highest activity among the six evaluated compounds followed by aloe-emodin.

For the *T. foetus* (bovine), Table 1 shows that in terms of IC_50_ value, chrysazin is 80.2 times more active than purpurin. The corresponding values of enhanced activity for the five other compounds, aloe-emodin, emodin, anthraquinone, anthrarufin, and rhein, are 37.8, 9.8, 8.6, 7.9, 5.6, respectively. For the bovine trichomonad strain, chrysazin exhibited the highest activity and the enhanced activities of emodin, anthraquinone, anthrarufin, and rhein are in the narrow 9.8–5.6 range.

We evaluated anthraquinone and its six derivatives against a range of non-pathogenic bacteria, i.e., commensals such as Lactobacillus spp. and pathogenic bacteria especially, those relevant to foodborne pathogens in a disc diffusion assay. None of the compounds were inhibitory for bacterial growth at the tested concentrations as compared to the antibiotic controls (Table 2, Table 3).

To evaluate if anthraquinone or its six derivative compounds have any activity against fungal pathogens, an agar diffusion assay was used for two pathogenic fungi. Whereas the positive control compound octyl gallate was inhibitory for the growth of both *A. fumigatus* AF293 and *C. albicans* ATCC10231, only emodin was active against pathogenic fungi and specifically against *Candida albicans* (Table 4).

### 2.1. Structure–Activity Relationships

Tested against the *T. vaginalis* G3 strain, these data show that the introduction of three phenolic hydroxyl (OH) groups into the 1, 2, 4 position of the right-hand benzene ring of the anthraquinone molecule (Figure 1) reduced activity because the anti-trichomonad activity of anthraquinone, which has no ring substituents, is 9.8-fold greater than that of purpurin, which has the three OH substituents. It seems that the three additional OH groups on purpurin resulted in an approximately 10-fold reduction in inhibitory activity. A similar enhancement of activity was observed with rhein, chrysazin, emodin, and aloe-emodin. The 166-fold enhanced activity of aloe-emodin compared to that of purpurin is striking. This compound contains two aromatic (phenolic) OH groups and one aliphatic hydroxymethyl OH group (Figure 1).

The structure–activity relationships for the other two trichomonad strains shown in Table 1 tend to parallel those of *T. foetus* strain C1, although the absolute enhancement values of all test compounds compared to purpurin differ somewhat. These observations suggest that there seems to be no direct relationships between the presence or absence of OH groups and relative activities.

The results with purpurin against the three trichomonads were unexpected and counterintuitive in view of the reported high activity of purpurin in numerous other biological activity studies highlighted below.

Table 1 shows, numerically, the trends of the active compounds at their respective IC_50_ values in relation to potencies and efficacy against the three pathogenic trichomonads. Significantly, similar growth inhibition values at IC_50_ values were obtained for each compound against the relevant trichomonad strain(s) tested. It would be of interest to determine if the in vitro results shown in Table 1 can be confirmed in vivo in farm and domestic animals and in humans.

### 2.2. Antifungal Activity of Emodin

None of the anthraquinone derivatives were effective against bacteria but emodin was selectively active against the yeast pathogen *C. albicans* at 5 mM. Percent zone of inhibition in *C. albicans* was determined as: 1) Negative control (DMSO only): 0%, 2) emodin: 76%, and 3) Positive control (Octyl gallate, 5 mM): 100%, respectively. The growth of the filamentous fungal pathogen *A. fumigatus* was not affected by emodin at the same test concentration.

The yeast pathogen *C. albicans* is one of the major agents causing human candidiasis, which includes vaginal infections. Yeast infection is continuously expanding globally due to the increased immune-compromised disorders [37]. While there have been seamless efforts to develop new antifungal drugs, current interventions have limited efficacy in treating fungal pathogens especially those resistant to conventional drugs [38]. Hence, the positive test with emodin warrants further clinical studies to confirm the beneficial in vitro results against *C. albicans*.

We found differential susceptibilities of the two pathogenic fungi (*A. fumigatus*, *C. albicans*) to growth inhibition by emodin. Of note, selective antifungal activity could also be found with the commercial antifungal drug fluconazole; the yeast pathogens *Candida* sp. and *Cryptococcus* sp. are susceptible to fluconazole, while the filamentous fungus *A. fumigatus* is not. This intrinsic resistance of *A. fumigatus* is linked to the naturally occurring T301I mutation in the cytochrome P450 enzyme gene encoding 14-α sterol demethylase A [39]. The precise determination of selective antifungal activity of emodin warrants future in-depth investigation.

### 2.3. Biological Activities of Purpurin that Impact Human Health

Purpurin has been reported to have numerous biological properties in cells and in rodents. These include the following observations. Tsang et al. [40] reported that purpurin inhibited *Candida dubliniensis* biofilm formation, suggesting that it has the potential to inactivate pathogenic fungi. The ability of purpurin to cross the blood–brain barrier and to ameliorate Alzheimer’s-disease-like symptoms in mice suggests its potential to treat sufferers of this uncurable neurological disease [41]. The reported amelioration of anxiety- and stress-induced depressive effects in the brains of mice suggests that purpurin has the potential to reduce the effects of depression via a serotonergic mechanism [42]. The reported potent in vitro anti-adipogenic effects in adipocyte cells and in vivo anti-obesity effects in mice consuming a high-fat diet suggest that purpurin has the potential to serve as a human anti-obesity agent [14]. The observed inhibition of adipocyte-derived leucine aminopeptidase and angiogenesis in zebrafish suggests that purpurin could also act as a new angiogenic agent [43]. The potent anti-oxidative and anti-inflammatory properties of purpurin in chemical and macrophage cell assays suggest the compound might be able protect foods and cells against oxidative damage and prevent in vivo oxidative stress and inflammation [13]. The inhibition of linoleic acid peroxidation by purpurin (% inhibition, concentration of purpurin): 84.8, 10 µM; 99.5, 50 µM; and 97.7, 100 µM. These values are greater than observed by 3-tert-butyl-4-hydroxyanisole (BHA), a synthetic antioxidant used to protect foods such as soybean oil against peroxidation. The reported purpurin-induced inhibition of carcinogenic heterocyclic amines in vitro [44,45] suggests that the molecule might also protect against the heat-induced formation of the carcinogens in cooked beef patties (hamburgers), an unsolved food safety issue described in detail elsewhere [46,47,48].

Some of these observations provide insights into the action of purpurin at the molecular-cellular level. The low anti-trichomonad activity of purpurin against the three strains observed here suggests that purpurin and the other evaluated anthraquinones act by unique mechanisms. Other studies that report on the antioxidant activities of anthraquinones in relation to their structure might provide insights into potential mechanisms of action of anthraquinones against trichomonads.

### 2.4. Anti-Oxidative Properties of Anthraquinones

Although we did not determine the antioxidative properties of the evaluated seven anthraquinones that might govern their anti-trichomonad mechanisms, it is instructive to mention reported relevant studies designed provide insight and to stimulate further needed studies. The relationship between the structure of a group of anthraquinones and their antioxidative activity was investigated by Nam, Kim, Nam and Friedman [13] in a study on the antioxidative and anti-inflammatory activities of four anthraquinones both in vitro and in cell assays: purpurin, (1,2,4-trihydroxyanthraquinone), anthrarufin (1,5-dihydroxyanthraquinone), chrysazin (1,8-dihydroanthraquinone), and anthraquinone, without OH substituents on the aromatic part of the molecule. Purpurin was the most active compound in all assays, as illustrated by the following micromolar IC_50_ values in the inhibition of the linoleic acid peroxidation assay: purpurin, 1.27, anthrarufin, 23.5, chrysazin, 202, and anthraquinone, 101. These results show that purpurin, with the lowest IC_50_ value (highest activity), is 159 times more active than chrysazin, which had the lowest activity. These anthraquinones differ only in the number and location of the OH groups attached to the aromatic anthraquinone molecule, ranging from zero for anthraquinone to two for anthrarufin and chrysazin, and three for purpurin.

In a related study, Zengin et al. investigated the antioxidative effects of the following three anthraquinones: alizarin, quinizarin, and purpurin [49]. Purpurin showed the highest activity. On the basis of these and related cellular and biochemical studies, the authors suggest that the three anthraquinones may be used as antioxidants in foods and in medicine. A computational study of four anthraquinones (aloe-emodin, chrysophanol, emodin, and 1,3,8-trihydroxyanthraquinone) by Marković, Jeremić, Dimitrić Marković, Stanojević Pirković and Amić [15] describes the molecular mechanisms through which stable anthraquinone free radicals are formed by abstracting the reactive free electrons from reactive oxygen species (ROS) in cells or from oxidized food fatty acids [15]. The theoretical results are consistent with a mechanism that suggests that the overall geometry of the anthraquinones that might govern antioxidative capacity is greatly affected by mutual interactions among different substituents, resulting in the formation of unique structural conformations that can interact with active sites of cells. Elsewhere, we also describe the antioxidative mechanisms of phenolic compounds present in potatoes [50] and computer modelling methods involving interaction of phenolic OH groups with artificial cell membranes designed to elucidate the antibiotic mechanisms of black tea theaflavins and green tea chatechins [51,52].

The cited studies facilitate our understanding of the mechanisms that govern the antioxidative and antibiotic properties of the three classes of natural phenolic compounds—anthraquinones, potato phenolics, and tea molecules. It might be of interest to apply these molecular, cellular, and computational methods to define the anti-trichomonad properties of the seven anthraquinones evaluated in the present study. It would also be of interest compare anti-trichomonad and other biological effects of anthraquinone glycosides such as sennoside and rhein-8-gluoside (Figure 1) to the corresponding aglycones without the carbohydrate side chains [53].

## 3. Materials and Methods

### 3.1. Materials

Anthraquinone and six derivatives with different substituents on the aromatic ring were obtained from Sigma-Aldrich (St. Louis, MO, USA). *Trichomonas vaginalis* human strain G3 was from Patricia Johnson, University of California at Los Angeles, CA, USA, *Tritrichomonas foetus* feline strain D1 was from Lynette Corbeil at the University of California at San Diego, CA, USA, and feline *Trichomonas foetus*-like organism (strain C1) from Stanley Marks, University of California at Davis, School of Veterinary Medicine, CA, USA. The pathogenic and nonpathogenic bacteria and pathogenic fungi were obtained from the in-house United States of Agriculture (USDA) collection or from the American Type Culture Collection (ATCC, Manassas, VA, USA).

### 3.2. Methods

The powdered anthraquinones were used to determine their inhibitory activities against: (a) three parasitic trichomonad strains (human *Trichomonas vaginalis* G3, feline *Tritrichomonas foetus*-like C1, and bovine *Tritrichomonas foetus* D1); (b) four pathogenic bacteria (*Salmonella enterica*, *Listeria monocytogenes*, *Staphylococcus aureus*, and *Bacillus cereus*); (c) four nonpathogenic bacteria (*E. coli* K12 used as replacement for pathogenic *E. coli*, *Lactobacillus acidophilus*, *Lactobacillus rhamnosus* GG, and *Lactobacillus reuteri*); and (d) two pathogenic fungi (*Aspergillus fumigatus* and *Candida albicans*).

#### 3.2.1. Stock Solutions of the Anthraquinone Powders

The powders were dissolved in dimethyl sulfoxide (DMSO) to either 100 mM (aloin and purpurin) or 10 mM (anthraquinone, anthrarufin, aloe-emodin, emodin, and chrysazin). Solutions were prepared fresh and vortexed immediately before use.

#### 3.2.2. Trichomonad Growth Inhibition Assays

Cultures of G3 strain of *T. vaginalis* and *T. foetus* C1 and D1 strains of were grown and maintained in 11 mL of TYM Diamond medium of pH 6.2. Every 24 h, the cells from C1, D1, and G3 strains were passed by inoculating 1000 μL of cells into a new 15 mL conical tube containing 10 mL of TYM Diamond medium. Then, the cells were incubated for 24 h at 37 °C. Inhibitory screens were carried out as previously described [33,34,35]. These assays were incubated at 37 °C for 24 h before being counted using a hemocytometer. Percentage inhibitory activities were calculated relative to the dimethyl sulfoxide (DMSO control). Stock solutions were diluted in media and tested over a range of increasing concentrations. IC_50_ concentrations were calculated from using non-linear curve fitting on the GraphPad software (San Diego, CA, USA) and the predicted IC_50_ concentration was confirmed by re-testing. All screening trials were performed a minimum of three times on three separate days to a standard error of ≤0.10.

#### 3.2.3. Bacterial and Fungal Screens with Anthraquinone Compounds

Anthraquinone derivatives were evaluated for their anti-bacterial in disc diffusion growth assays at 100 μM (aloin and purpurin) or 10 μM (anthraquinone, anthrarufin, aloe-emodin, emodin, and chrysazin) with the DMSO (negative control) diluted 1:1000 in media to have the same DMSO vehicle control concentration as the test powders. Positive controls for growth inhibition for the various bacteria were discs containing the following antibiotics: levofloxacin 5 μg, gentamicin 10 μg, and gentamicin 120 μg. None of the compounds had anti-bacterial activity at the concentrations tested. The antifungal activity of anthraquinone and its derivatives was examined in *Aspergillus fumigatus* AF293, a causative agent for invasive aspergillosis, and *Candida albicans*. Stock compounds were diluted to either 20 mM (aloin and purpurin) or 5 mM (anthraquinone, anthrarufin, aloe-emodin, emodin, and chrysazin) for fungi, respectively. DMSO was used as a negative vehicle control and 5 mM octyl-gallate was used for the positive control for growth of both fungi. In both *A. fumigatus* and *C. albicans* tests, 5 μL of anthraquinones were spotted onto the lawn of fungi (in duplicate), which were grown on Potato Dextrose Agar (PDA) or Yeast Peptone Dextrose (YPD; Bacto yeast extract 1%, Bacto peptone 2%, glucose 2%) (Millipore Sigma, St. Louis, MO, USA) for *A. fumigatus* or *C. albicans*, respectively. Fungi were incubated at 35 °C and the formation of zones of inhibition in millimeters (mm) were monitored at 24 and 48 h.

## 4. Conclusions

The evaluated test compounds were found to be active against the three parasitic protozoa; emodin also possessed antifungal activity against the yeast pathogen *C. albicans*. None of the compounds showed activity against pathogenic foodborne bacteria or lactobacilli. It seems, the nature and number of substituents on the anthraquinone ring were found to influence the wide-ranging relative potencies, as determined from IC_50_ values. In our previous study by Liu et al. [34], we reported on the IC_50_ values of the widely used drug metronidazole for each of the three trichomonads: *T. vaginalis* G3 = 0.72 µM; *T. foetus* C1 = 0.55 µM; *T. foetus* D1 = 0.49 µM. The data in Table 1 show that the corresponding values of aloe-emodin against the three trichomonads (IC_50_ = 0.6109, 1.41, and 2.47) are of the same order of magnitude as those of metronidazole. Because of high rates of clinical resistance to the widely used synthetic drug metronidazole mentioned earlier, new approaches are needed to complement the available therapeutic treatments [32]. The most active anthraquinone compounds, aloe-emodin and chrysazin for *T. vaginalis* (human) and chrysazin and aloe-emodin against both the farm and domestic animal trichomonads, merit further study to define their ability to ameliorate both trichomoniasis in rodents [16] and humans [54,55] and trichomoniasis in farm and domestic animal against nonresistant as well as resistant trichomonad strains. In vivo animal and human studies should determine the ratio of effective to toxic doses. Finally, the results of the present anti-trichomonad studies and the cited studies by other investigators on health benefits of anthraquinones suggest that that this class of natural compounds has the potential to ameliorate multiple diseases.

## Figures and Tables

**Table 1:** Activity of anthraquinone and derivatives against pathogenic trichomonads. The table shows the calculated IC_50_ for each anthraquinone and substituted derivative and its corresponding percent growth inhibition at the designated concentration *^a^*.

	*T. vaginalis* G3 (human)			*T. foetus* C1 (Feline)			*T. foetus* D1 (Bovine)		
Compound	IC_50_ (µM)	Percent Inhibition (%)	Percent Error (%)	IC_50_ (µM)	Percent Inhibition (%)	Percent Error (%)	IC^50^ (µM)	Percent Inhibition (%)	Percent Error (%)
Purpurin	101.60(1.0) *^b^*	51.2	2.5	133.10(1.0)	50.29	0.59	78.62(1.0)	49.64	0.72
Anthraquinone	10.43 (9.8)	50.7	1.4	25.55(5.2)	45.6	8.8	9.19(8.6)	51.4	2.9
Anthrarufin	ND	ND	ND	8.10(16.4)	46.1	7.8	7.10(7.9)	45.4	9.1
Chrysazin	8.90(11.4)	52.9	5.7	0.979(137.8)	47.4	5.3	0.98(80.2)	53.1	6.3
Aloe-Emodin	0.6109(166.2)	47.9	4.1	1.41(94.4)	52.2	4.4	2.47(37.8)	50.36	0.72
Emodin	ND	ND	ND	ND	ND	ND	8.00(9.8)	48.0	4.0
Rhein w/HPLC	5.06(20.1)	53.3	6.5	21.00(33.8)	52.2	4.4	14.04(5.6)	51.9	3.9

*^a^* Compounds that did not show >90% inhibition in preliminary screenings were not titrated (ND, not determined) for IC_50_ determination. All predicted IC50 values were re-tested and confirmed. Results shown were the average of three independent experiments with calculated percent error and IC_50_ values. *^b^* Numbers in parenthesis are relative activities (potencies) in terms of IC_50_ values.

**Table 2:** Table depicts the zones of inhibition in mm (growth inhibition) from a disc diffusion assay targeted against bacterial pathogens using DMSO vehicle control, antibiotic controls, and test compounds at indicated concentrations *^a,b^*. *^a^* at 100 μM, *^b^* at 10 μM.

Compound	*Salmonella* *Enterica*	*Listeria* *Monocytogenes*	*Staphylococcus Aureus*	*Bacillus Cereus*
DMSO	0	0	0	0
Levofloxacin 5 µg	30	17	36	26
Gentamicin 10 µg	16	22	25	15
Gentamicin 120 µg	22	30	30	20
Purpurin *^a^*	0	0	0	0
Anthraquinone *^a^*	0	0	0	0
Anthrarufin *^b^*	0	0	0	0
Chrysazin *^b^*	0	0	0	0
Aloe-Emodin *^b^*	0	0	0	0
Emodin *^b^*	0	0	0	0
Rhein w/HPLC *^b^*	0	0	0	0

**Table 3:** Table depicts the zones of inhibition in mm (growth inhibition) from a disc diffusion assay targeted against bacterial commensals using DMSO vehicle control, antibiotic controls, and test compounds at indicated concentrations *^a,b^*. *^a^* at 100 μM, *^b^* at 10 μM.

Compound	*Escherichia coli* K12	*Lactobacillus Acidophilus*	*Lactobacillus Rhamnosus* GG	*Lactobacillus Reuteri*
DMSO	0	0	0	0
Levofloxacin 5 µg	30	0	15	5
Gentamicin 10 µg	18	8	8	11
Gentamicin 120 µg	20	15	15	20
Purpurin *^a^*	0	0	0	0
Anthraquinone *^a^*	0	0	0	0
Anthrarufin *^b^*	0	0	0	0
Chrysazin *^b^*	0	0	0	0
Aloe-Emodin *^b^*	0	0	0	0
Emodin *^b^*	0	0	0	0
Rhein w/HPLC *^b^*	0	0	0	0

**Table 4:** Activity of anthraquinone and derivatives against pathogenic fungi. The table shows the calculated zone of inhibition (mm) for each anthraquinone and substituted derivative at the designated concentration ^a^.

Compound	Concentration (mM)	*A. fumigatus* AF293	*C. albicans* ATCC10231
Aloin	20	0.00	0.00
Purpurin	20	0.00	0.00
Anthraquinone	5	0.00	0.00
Anthrarufin	5	0.00	0.00
Chrysazin	5	0.00	0.00
Aloe-Emodin	5	0.00	0.00
Emodin	5	0.00	1.10 (76%) ^c^
Octyl gallate	5	1.15 (100%) ^c^	1.45 (100%) ^c^
DMSO	50 ^b^	0.00 (0%) ^c^	0.00 (0%) ^c^

^a^ Data shown were the average of duplicated results with calculated zone of inhibition values. ^b^ Percent (%) concentration; negative control. ^c^ Numbers in parenthesis (%) are relative zone of inhibition compared to the positive control (octyl gallate), which is 100%.
